# Expression and significance of IL-6 and IL-8 in canine mammary gland tumors

**DOI:** 10.1038/s41598-023-28389-3

**Published:** 2023-01-24

**Authors:** Xiaoli Ren, Yuying Fan, Dongmei Shi, Yun Liu

**Affiliations:** 1 Zhengzhou City Key Laboratory of Animal Nutritional Metabolic and Poisoning Diseases, College of Veterinary Medicine, Henan University of Animal Husbandry and Economy, Zhengzhou, 450046 China; 2 Heilongjiang Provincial Key Laboratory of Pathogenic Mechanism for Animal Disease and Comparative Medicine, Heilongjiang Key Laboratory for Laboratory Animals and Comparative Medicine, Department of Veterinary Surgery, College of Veterinary Medicine, Northeast Agricultural University, Harbin, 150030 China

**Keywords:** Breast cancer, Diagnostic markers, Predictive markers

## Abstract

Mammary gland tumors are the most common malignant diseases which seriously threaten the health of women and female dogs. There is a lack of an effective tumor marker which can effectively assist in the early diagnosis, and prognosis of mammary gland tumors in veterinary clinical medicine. IL-6, and IL-8 as immunosuppressive factors may stimulate tumor cells growth, contribute to loco-regional relapse and metastasis that might be utilized as a marker for immunity status and monitoring of the course of tumor. The present study aimed to investigate the expression of serum/tissue IL-6, IL-8 and IL-10 in canine mammary gland tumors using Enzyme linked immunosorbent assay(ELISA), Western blot and Immunohistochemistry assay(IHC) to determine whether it is associated with tumor progression. The results showed that levels of IL-6, IL-8 and IL-10 in serum were higher in malignant tumor group than that in benign tumor and control group; the expression levels of IL-6 and IL-8 were significantly elevated in grade III than in grade I and II and was related to metastasis. Likewise, IL-6 and IL-8 were also highly expressed in malignant tumor tissues. Elevated expression of IL-6 was associated with histopathological grade and metastases in malignant tumors. Moreover, high expression of IL-6 occurred in the Basal-like subtypes whereas high expression of IL-8 occurred in the Luminal B subtypes. The results of this study indicated that changes of IL-6 and IL-8 in the tumor microenvironments were closely related to the diseases status and may be used as a potential diagnostic or biomarker in canine mammary gland tumors.

## Introduction

Mammary gland tumors are the most common heterogeneous disease which seriously threatens the health of female dogs, approximately 50% of these are considered malignant by histopathologic diagnosis^1,2^. The inflammatory responses have an multifaceted role in mammary carcinogenesis, contributing to tumor evasion of surveillance, matrix remodeling and angiogenic switch, acquisition of different hallmark capability (tumor growth, invasion and metastasis et al*.*)^3^. Moreover, tumor microenvironment contains an abundance of cytokines, which contribute to multiple hallmark capabilities of the tumorigenesis by recruiting inflammatory cells to the tumor microenvironment, further amplifying inflammation, promoting tumor proliferation, metastasis, angiogenesis, lymphangiogenesis, and poor prognosis in different types of cancers, and leading to immune system dysfunction^4–7^. Therefore, these inflammation-related cytokines may be used as biomarkers for detecting progression of malignant tumor, tumor metastasis, as well as being potential therapeutic targets.

Interleukins (ILs) are cytokines that mediate leukocyte crosstalk and modulate proliferation, differentiation, growth, survival and functions of immune cells^8,9^. Cytokines such as IL-6, IL-8 and IL-10 are produced and secreted by activated immune cells such as macrophages, monocytes and lymphocytes, as well as by many cancer cell types^10,11^. These cytokines act in an autocrine or paracrine manner and promote cancer cell infiltration, metastasis and acute phase responses in many cancers^12,13^. In addition to their role in immunity, IL-6, IL-8 and IL-10 promote growth, invasion, and metastasis and drug resistance in various tumor types^6,14^. Low serum IL-6 levels enhance immune responses and inhibit the growth of cancer cells, whereas high serum IL-6 levels decrease immunity and enhance infiltration by tumor cells. High serum IL-6 levels correlate with tumor stage and poor survival^15^. Tumor and tumor-associated immune cells secrete IL-8, which promotes angiogenesis, invasion and metastasis of cancer cells^16,17^. IL-10 is an immunosuppressive cytokine, which inhibits the proliferation and differentiation of Th-1 and Th-2 cells and their ability to produce and secrete IL-2 and IFN-γ. It diminishes anti-tumor responses by inhibiting the activities of monocytes and NK cells^11,18^. In animal model studies, induced expression of IL-10 in breast cancer cells reduces tumor growth^19^. IL-10 knockout mice show prolonged survival and increased rejection of bladder tumor cells, indicating that higher IL-10 levels facilitate tumor immune escape^20^. However, the status of IL-6, IL-8 and IL-10 expression in canine mammary gland tumor tissues and serum has not been established. Levels of estrogen receptor (ER), progesterone receptor (PR) and epidermal growth factor receptor 2 (Her-2) are gold standards for predicting survival and treatment responses in human breast cancer patients^21^. But the correlation between the cytokine levels in peripheral blood and the tumor tissue microenvironment with the PR, ER and Her-2 status of the mammary gland tumors is unknown. We therefore analyzed the relationship between the levels of IL-6, IL-8 and IL-10 cytokines in the serum and tumor tissues, and the molecular subtypes of dogs with canine mammary gland tumors (CMGTs).

## Results

### General characteristics of CMGTs and control female dogs

The general clinical and pathological characteristics of female dogs with CMGTs and controls are summarized in Table 1. A total of 112 female dogs with CMGTs and 38 healthy female dogs with no tumor history or inflammation were used in the present study. Most of the dogs have not undergone ovarian hysterectomy. The age of control female dogs ranged from 3 to 14 years old, with an average age of 8.2 ± 2.6 years old. The body weight of the control female dogs ranged from 4 to 43 kg, with an average body weight of 9.2 ± 4.23 kg. The age of tumor-laden female dogs ranged from 4 to 16 years old, with an average age of 10.2 ± 5.6 years old. The body weight of tumor-laden female dogs ranged from 6 to 46 kg, with an average body weight of 14.2 ± 6.04 kg. The average age and body weight of the CMGTs and control groups of female dogs were similar (*P* > 0.05). The average age of female dogs with malignant and benign mammary gland tumors were 10.2 and 8.7 years, respectively. There were 28 (53.84%) cases of simple carcinoma and 18 (30.00%) cases of fibroadenoma.

**Table 1:** General clinical and pathological characteristics of female dogs with CMGTs.

Characteristics	Number	Frequency (%)
Control group	38	
Ages		
≥ 8	20	52.63
< 8	18	47.37
Benign tumor group	60	
Ages		
≥ 8	32	53.33
< 8	28	46.67
Size (cm)		
T1 (< 3)	26	43.33
T2 (3 ≤ T ≤ 5)	20	33.33
T3 (> 5)	14	23.33
Histrogical type		
Fibroadenoma	18	30.00
Intraductal papillary adenoma	13	21.67
Simple adenoma	4	6.67
Hyperplasia/dysplasia(duct ectasia, lobular hyperplasia and epitheliosis)	19	31.66
Others	6	10
Malignant tumor group	52	
Ages		
≥ 8	18	34.62
< 8	34	65.38
Size (cm)		
T1 (< 3)	8	15.38
T2 (3 ≤ T ≤ 5)	27	51.92
T3 (> 5)	17	32.69
Histrogical type	52	
Simple carcinoma	28	53.84
Solid carcinoma	12	23.07
Intraductal papillary carcinoma	5	9.62
Invasive micropapillary carcinoma	2	3.85
Carcinoma in situ	2	3.85
Others carcinoma	3	5.77
Histologic grade		
I	16	30.77
II	24	46.15
III	12	23.08
Molecular subtype		
Luminal A	14	26.92
Luminal B	24	46.15
Her-2 positive	8	15.38
Basal-like	6	11.54

### Relationship between levels of serum IL-6, IL-8 and IL-10 in serum and clinicopathological characteristics with CMGTs

**Figure 1:**
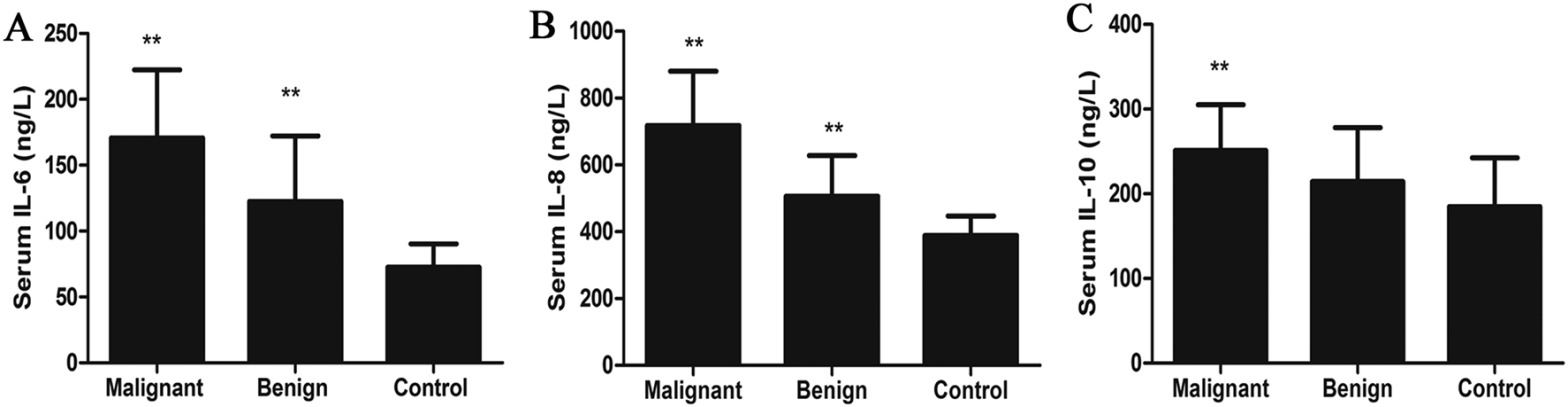
Serum levels of IL-6, IL-8 and IL-10 in CMGTs and healthy dogs. The serum levels of IL-6 (**A**), IL-8 (**B**) and IL-10 (**C**) in CMGTs and healthy dogs were detected by ELISA assay. One-way analysis of variance (ANOVA) by Tukey’s multiple comparison test was used to analyze multiple group comparisons data. The results from three independent experiments are presented as the mean ± standard deviation (SD). *denotes *P* < 0.05 and **denotes *P* < 0.01 compared with the healthy canine mammary gland serum group.

The median serum IL-6, IL-8 and IL-10 level in the 38 healthy female dogs was 73.47 ng/L (95% CI 66.78–78.38) with a range from 42.8 to 107.9, 377.8 ng/L (95% CI 369.2–407.6) with a range from 308.8 to 508.1 and 166.3 (95% CI 165.8–203.8) with a range from (113.1–299.2), respectively. The serum IL-6, IL-8, and IL-10 levels were significantly higher in the malignant CMGTS group than that of benign CMGTs and the control group (*P* < 0.05; Fig. 1). However, serum IL-10 levels were similar in the benign CMGTs and the control groups (*P* > 0.05; Fig. 1). There was no correlation between the serum IL-6, IL-8 and IL-10 levels and age or tumor siz**e** (Table 2). However, canines with metastasis showed higher serum IL-6 levels (*P* = 0.0251; Table 2). The high serum IL-6 and IL-8 levels were associated with the higher histological grade and malignant CMGTs. Serum IL-10 levels did not correlate with the molecular subtypes. The serum IL-6 levels were higher in the basic-like subtype CMGTs, whereas, serum IL-8 levels were higher in the Luminal B subtype CMGTs. This suggests that serum IL-6 and IL-8 levels correlate with the molecular subtypes of CMGTs.

**Table 2:** Relationship between serum IL-6, IL-8, and IL-10 levels and clinicopathological characteristics in female dogs with malignant CMGTs.

Characteristics	N	IL-6 (ng/L)	*P*-value	IL-8 (ng/L)	*P*-value	IL-10 (ng/L)	*P*-value
Ages							
≥ 8	18	159.5 ± 46.14	0.4195	727.1 ± 161.7	0.5919	255.2 ± 49.69	0.3998
< 8	34	171.3 ± 51.4		701.4 ± 166.6		242.6 ± 52.6	
Size (cm)							
T1 (< 3)	8	141.1 ± 34.82	0.0938	685.2 ± 148	0.7805	240.6 ± 39.46	0.5671
T2 (3 ≤ T ≤ 5)	27	168.5 ± 48.01		717.5 ± 155.3		247.4 ± 47.69	
T3 (> 5)	17	188.4 ± 58.42		735.0 ± 185.1		261.2 ± 59.87	
Metastasis state							
Yes	12	199.7 ± 54.76	*0.0251	756.3 ± 157.2	0.3599	275.7 ± 39.3	0.0509
No	40	162. ± 47.9		706.8 ± 163.9		243.4 ± 51.57	
Histologic grade							
I	16	144 ± 42.72	^#^0.0014	567.9 ± 101.3	^#^0.0001	221 ± 42.56	**0.0015
II	24	165.8 ± 46.74		746.7 ± 121.5		252.5 ± 45.57	
III	12	216.6 ± 43.21		861.8 ± 141.5		287.4 ± 47.8	
Molecular subtype							
Luminal A	14	169.5 ± 41.53	**0.008	708.1 ± 145.9	^#^0.0463	246.3 ± 59.21	0.1534
Luminal B	24	165.7 ± 53.8		775.4 ± 174.6		265.5 ± 45.94	
Her-2 positive	8	142.6 ± 31.03		619.3 ± 105.9		227.0 ± 45.63	
Basal-like	6	231.8 ± 45.7		645.2 ± 137.9		234.7 ± 45.04	

*Denotes unpaired *t* test; **denotes One-way ANOVA test; ^#^denotes Kruskal–Wallis; *P* < 0.05 was considered to be statistically significant.

### Relationship between the tissue expression of IL-6, IL-8, IL-10 and clinicopathological factors in CMGTs

**Figure 2:**
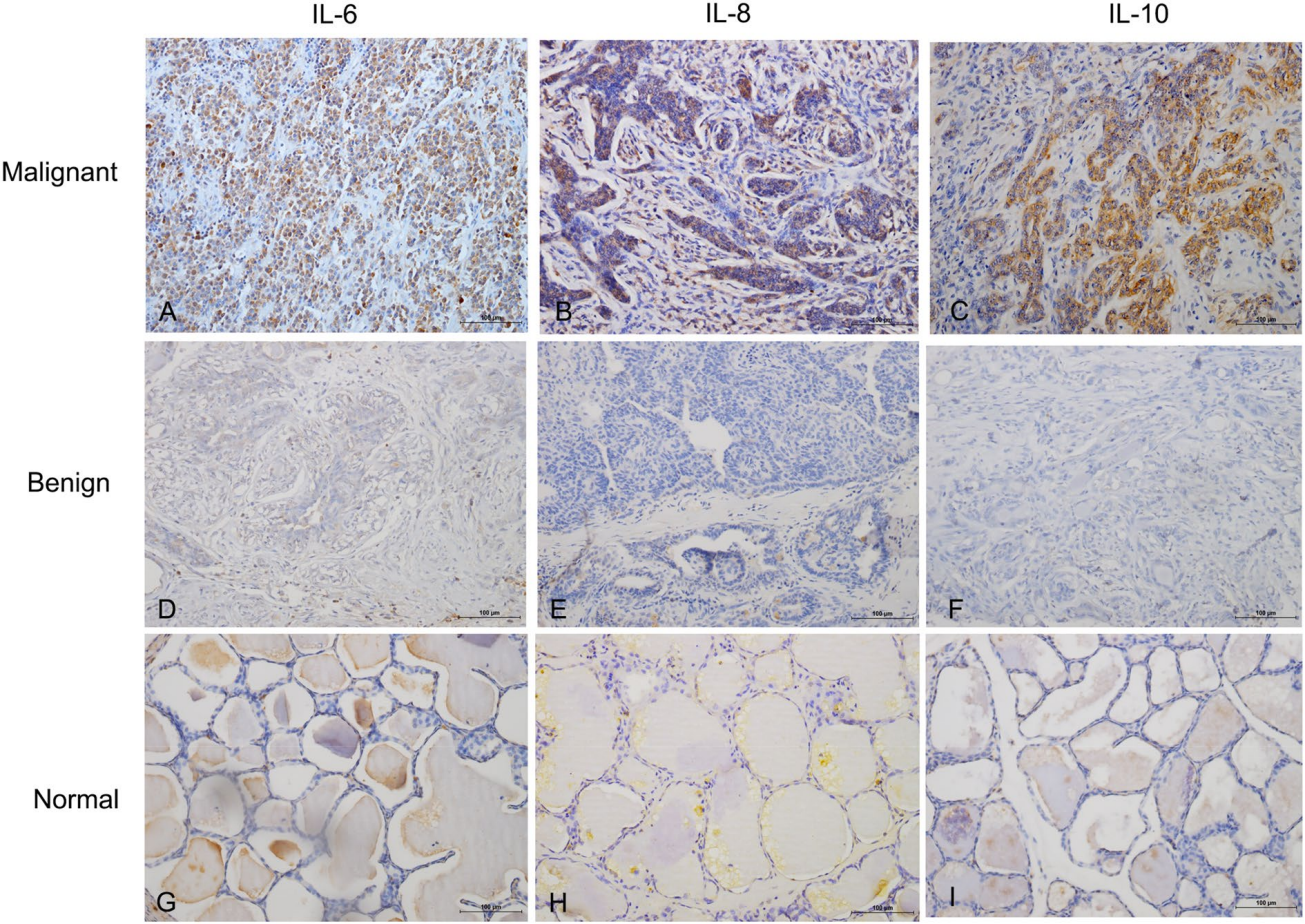
Immunohistochemical staining of IL-6, IL-8 and IL-10 in CMGTs and normal canine mammary gland tissues. Representative images (200X) show IHC staining of (**A**, D and **G**) IL-6, (**B**, **E** and **H**) IL-8 and (**C**, **F** and **I**) IL-10 in CMGTs and normal canine mammary gland tissue sections. Both cell membrane and cytoplasmic staining is indicated.

**Figure 3:**
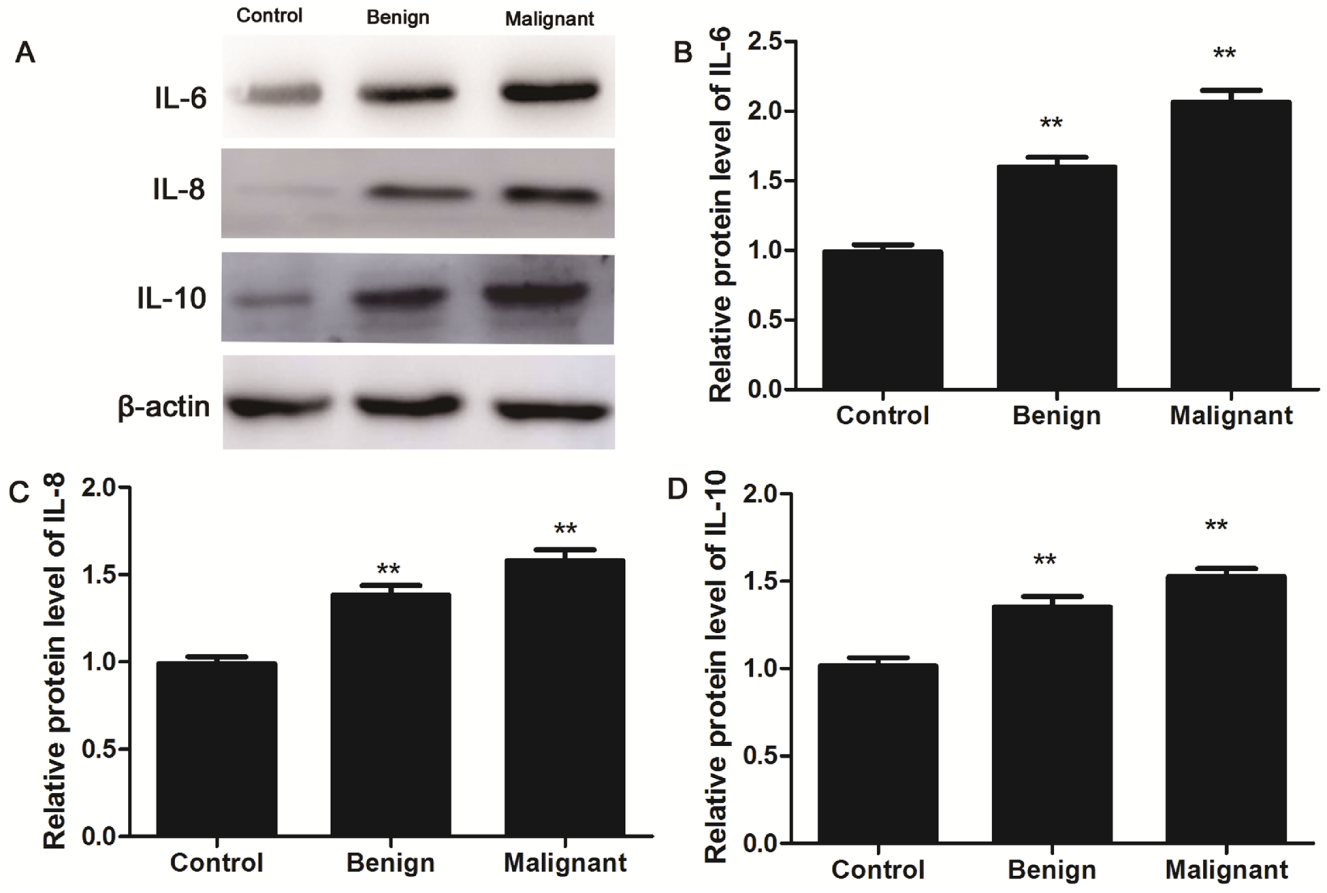
Expression of IL-6, IL-8 and IL-10 proteins in CMGTs and normal canine mammary gland tissues. (**A–D**) The levels of IL-6, IL-8 and IL-10 protein in CMGTs and normal canine mammary gland tissues were estimated by western blot analysis. β-actin was used as loading control. Uncropped images for Western blots are presented in Supplementary Fig. S1. Relative protein levels were quantified using ImageJ 1.48 software. Immunoblots were captured to calculate the normalized values presented in the graph by estimation of express levels from IL-6, IL-8 and IL-10 proteins relative to β-actin protein. Data are presented as mean ± SD of triplicate experiments. *denotes *P* < 0.05 and **P < 0.01 compared with the healthy canine mammary gland tissues group.

Next, we analyzed the expression of IL-6, IL-8, and IL-10 for the CMGTs samples collected from the veterinary clinical hospital by IHC assay. Brown or yellow colour indicated positive IL-6, IL-8 and IL-10 staining on the cell membrane or in the cytoplasm of tumor cells (Fig. 2). Among the 52 malignant CMGTs, 35 (67.3%), 40 (76.9%), and 22 (42.3%) samples were positive for IL-6, IL-8 and IL-10 expression, respectively. Among the benign CMGTs, 13 (21.66%), 11 (18.3%) and 10 (16.67%) were positive for IL-6, IL-8 and IL-10 expression, respectively. The expression of IL-6 and IL-8 was significantly higher in the malignant CMGTs tissues than in control and benign CMGTs tissues (*P* = 0.000; Table 3). Western blot analysis further confirmed high levels of IL-6 and IL-8 protein expression in malignant CMGTs tissues than the benign CMGTs tissues (Fig. 3). Based on the above IHC results, we then performed correlation analysis of the expression IL6, IL-8 and IL-10 cytokines with the clinicopathological features. IL-8 and IL-10 expression in the malignant CMGTs tissues did not correlate with age, tumor size, tumor metastasis (*P* > 0.05; Table 4). Moreover, IL-6 and IL-8 expression in the CMGTs positively correlated with the histological grade, whereas, IL-6 expression in the CMGTs positively correlated with tumor metastasis (*P* < 0.05; Table 4). This suggested that IL-6 and IL-8 expression were closely related to tumor metastasis and the degree of malignant CMGTs. Moreover, the expression of cytokines is also associated with molecular subtypes, and the positive expression of IL-6 in the basal type (Basal-like type) (5/6, 83.33%) is high, and the positive expression of IL-8 in Luminal B is high. These data are in accordance with the serological results, indicating that the expression of IL-6 and IL-8 is related to the occurrence of breast tumor in dogs, and its expression is related to the molecular subtype. These data suggest IL-6 and IL-8 expression in the CMGTs are associated with the molecular subtypes of CMGTs.

**Table 3:** The expression of IL-6，IL-8 and IL-10 in CMGTs and normal canine mammary gland tissues detected by immunohistochemistry.

Groups	n	IL-6		IL-8		IL-10	
		−	+ – +++	−	+–+++	−	+– ++
Malignant tumor group	52	17	35	12	40	30	22
Benign tumor group	60	50	13	42	11	50	10
Control group	38	38	0	38	0	38	0
χ^2^		51.934		64.779		24.715	
*P*值		*0.000		*0.000		*0.000	

*denotes *P* < 0.05 compared with the control group.

**Table 4:** Relationship between the expression of IL-6, IL-8, IL-10 protein and clinicopathological factors in malignant CMGTs tissue.

Characteristics	n	IL-6			IL-8			IL-10		
		n (%)	χ^2^	*P*	n (%)	χ^2^	*P*	n (%)	χ^2^	*P*
Years										
≤ 8	18	10 (55.56%)	1.728	0.189	12 (66.67%)	1.631	0.202	6 (33.33%)	1.821	0.177
> 8	34	25 (73.53%)			28 (82.35%)			18 (52.94%)		
Size (cm)										
T1(< 3)	8	3 (37.5%)	5.017	0.081	6 (75%)	0.223	0.894	4 (50%)	0.139	0.933
T2(3 ≤ T ≤ 5)	27	22 (78.57%)			23 (82.14%)			12 (42.85%)		
T3((> 5)	17	10 (71.42%)			11 (78.57%)			6 (42.85%)		
Node state										
Yes	12	11 (91.67%)	4.207	*0.040	8 (66.67%)	0.924	0.336	7 (58.33%)	1.641	0.200
No	40	24 (60%)			32 (80%)			15 (37.5%)		
Histologic grade										
I	16	6 (37.5%)	6.888	*0.032	9 (56.25%)	6.816	0.033	5 (31.25%	2.068	0.356
II	24	19 (79.16%)			22 (91.67%)			10 (41.67%)		
III	12	10 (83.33%)			9 (75%)			7 (58.33%)		
Molecular subtype										
Luminal A	14	8 (57.14%)	9.053	*0.029	12 (85.71%)	10.31	*0.016	5 (35.71%	3.57	0.312
Luminal B	24	19 (79.16%)			21 (87.5%)			14 (58.33%)		
Her-2 positive	8	2 (25%)			3 (37.5%)			2 (25%)		
Basal-like	6	5 (83.33%)			4 (66.67%)			2 (33.33%)		

*P < 0.05 was considered to be statistically significant.

## Discussion

Malignant mammary gland tumors are highly prevalent in canines, which serve as human companions and share similar living environments^1^. Spontaneous CMGTs as translation models for studying human breast cancer, because of their similar features in epidemiological data, prognostic factors and histological patterns of the neoplastic, such as the relative age of onset, risk factors, histological and molecular features, biological behavior, metastatic patterns, and therapeutic responses^1,22^. Our study shows that simple carcinomas are a common subtype of malignant CMGTs, whereas, the most common benign CMGTs are fibroadenomas. Moreover, higher morbidity and malignancy rates are observed in older canines.

Inflammation modulates the occurrence and development of malignant tumors^23^. Cytokines are low molecular weight glycoproteins that modulate the intensity and duration of immune response by regulating the proliferation, and differentiation of their target immune cells. Cytokines play diverse roles in cancer initiation and progression^14,23^. Elevated serum IL-6 levels in a large number of patients with progressive metastatic breast cancer (41/65; 63%) are concerned to poor event-free survival (EFS), overall survival (OS), and increased risk of early recurrence and bone metastasis^4^. IL-6 levels involved in clinical tumor stages and pathological grades in breast cancer; high IL-6 levels indicate poor prognosis in breast cancer patients^15,24,25^. However, IL-6 expression was related to OS, and unrelated to lymph node metastasis, tumor size, histological grade or DFS in patients with breast cancer^26^. In CMGTs, IL-6 levels in malignant and metastatic tumor tissues are 5–sixfold higher than that in benign tumors (3.75% vs. 0.65%)^27^. Zakrzewska et al. demonstrated that serum IL-8 levels were significantly higher in malignant breast cancer patients than in the benign breast cancer patients and healthy controls; high serum IL-8 levels were related to tumor staging^28^. IL-8 levels are low in healthy and benign breast tumor tissues and high expression of IL-8 in breast cancer tissues has nothing to do with age and clinical stages, but correlated with the histological type (invasive and non-invasive carcinomas) and lymph node metastasis, thereby distinguishing benign and malignant breast tumors^16,17,29^. IL-10 expression was decreased in simple adenoma and mixed carcinoma tissues and increased in intraductal papillary carcinoma tissues^5^. Moreover, serum IL-10 levels were decreased in patients with benign mixed carcinoma without metastasis (MC-BMT); benign tumors with hyperplasia and intraductal papillary carcinoma show higher levels of IL-10 than the normal mammary glands^30^. Although serum IL-10 levels were increased in invasive ductal carcinomas, they had nothing to do with tumor grading^31^. The serum IL-10 levels are higher in the breast cancer patients than that in healthy females, and further increased with cancer progression^32^. Higher serum IL-10 levels in breast cancer patients correlate with the clinical stages and metastatic cancer^33^. In canines, serum and tissue of IL-8 and IL-10 levels are higher in the dogs with inflammatory mammary cancer (distant metastases) than in the dogs with non-inflammatory mammary cancer malignant mammary tumors^30^. The plasma concentrations of IL-8 was significantly increased in female dogs with non-metastatic and metastatic malignant mammary gland tumors compared to the healthy dogs; moreover, the plasma concentrations of IL-8 was significantly higher in the dogs with grade 3 tumors compared to that dogs with grade 1 and grade 2 tumors^34^. Consistent with previous findings, our study showed higher expression of IL-6 and IL-8 in the serum and tissues of dogs with malignant CMGTs than that in the dogs with benign CMGTs. We observed higher IL-10 expression in 22 out of 52 malignant CMGTs. We also demonstrated that serum IL-6 levels were associated with histological grade and lymph node metastases. Moreover, IL-6 and IL-8 are highly expressed on the cell membrane or in the cytoplasm of malignant tumor cells. The expression of IL-6, IL-8, and IL-10 in the CMGTs tissues was consistent with their serum levels. High expression of IL-6 and IL-8 correlated with the malignancy and metastasis of CMGTs, and therefore can be used as candidate biomarkers for tumor diagnosis.

The relationship between inflammatory cytokines and breast cancer molecular subtypes has not been well established. IL-6 and IL-8 levels are related to ER and Her-2 antigen expression in patients with ductal carcinomas; ER^+^ Her2^-^ breast cancer patients are associated with higher serum IL-6 levels than the ER^-^ Her-2^+^ breast cancer patients; serum IL-6 and IL-8 levels are independent of PR expression in the breast cancer patients^15,25^. However, the relationship between ER status and IL-8 levels is controversial. IL-8 expression is inversely correlated with ER status, but positively correlated with estradiol; moreover, breast cancer patients with high levels of IL-8 during the early stages are in relation to shorter relapse-free survival (RFS), whereas patients with low IL-8 levels are associated with ER^−^ PR^−^ and Her-2^−^ Her-2^+^ phenotypes^35^. Higher IL-6 levels are observed in breast cancer patients with the − 174 G/G genotype, ER negative tumors and bone metastasis^36^. A study of 105 breast cancer patients showed that IL-10 expression was higher in patients with higher ER negative and SBR classification; higher serum IL-6 levels were more common in triple negative breast cancer (TNBC) patients and used to monitor predictive therapeutic response^4^. In this study, higher IL-6 expression was observed in Basal-like (ER^-^/ PR^-^ /Her-2^−^) subtype, whereas, higher IL-8 expression was relevant to the Luminal B subtypes. Studies have suggested that IL-6 and IL-8 expression may be utilized as potential diagnosed and immune therapy biomarker of CMGTs^37,38^.

Inclusion, this present study demonstrates that expression level of IL-6 and IL-8 in CMGTs are increased, which makes the body’s immune regulation imbalance, and is significantly associated with the severity of disease. It has very important clinical value for understanding the condition, observing the efficacy and prognostic evaluation.

## Materials and method

### Ethical statement

This study was the performed in accordance with a protocol approved by the Institutional Ethics Committee in the Use of Animal of the Northeast Agricultural University, school of Veterinary Medicine, Northeast Agricultural University. China (protocol:SRM-11). Tissue samples and peripheral blood samples were obtained from dogs undergoing standard of care surgical procedure removal of mammary tumors and the jugular or lateral saphenous vein, respectively. Signed informed consents were obtained from all the owners of dogs involved in the present study, which were with regard to standard-of-care veterinary diagnostics and treatment and use of excess tissues for research purposes. All experiments methods were carried out in accordance with relevant guidelines and complies with ARRIVE 2.0 guidelines^39^.

### Sample collection and clinical features

150 female dog samples (112 CMGTs and 38 healthy controls) were collected at the College of Veterinary Medicine, Northeast Agricultural University, Harbin, China. Metastasis status was confirmed by detailed imaging examination, needle aspiration cytology as well as Histopathological evaluation. None of patients had received chemotherapy, radication therapy or other anti-tumor therapies before and or after surgery. Canine clinicopathological parameter in the present study were obtained through clinical veterinarian, such as: age, breed, spay status, medical history, times of pregnancy/bearing birth, tumor size, et al. The histological diagnosis of tumor tissue samples was performed according to the classification proposed by Goldschmidt et al.^40^. Histological Grade of Malignancy tumor were based on a 2013 report by Peña et al.^41^.

### Evaluation of immunohistochemistry staining

After surgical removal, one portion of tumor tissues were frozen in liquid nitrogen and stored at − 80 °C. The remaining portion of tumor tissues were fixed in 10% buffered formalin, paraffin embedded and cut into 3 μm thick sections. Immunohistochemical method (IHC) was performed on 3 μm thick sections with the following primary antibodies, such as: polyclonal rabbit anti-IL-6 (dilution, 1:200), polyclonal rabbit aniti-IL-8 (dilution, 1:200) and polyclonal rabbit anti-IL-10 (1:150) (Bioss Biotechnology, Beijing, China). Negative control were incubated with PBS replacing the primary antibody to confirm the specificity of the antibody, and adjacent normal canine mammary tissue was used as internal positive control. The tissues IHC staining result was analyzed according to the semi-quantiatively analysis. After excluding areas of nonspecific staining, the percentage of immuno-positive cells was obtained from 10 random high-powered field images per section, under a 40 × objective magnifification. Samples were evaluated for staining intensity, as follows: (−) none, ( +)weak staining, (+ +)diffuse staining, (+ + +)strong staining. The percentage of positive cells was set as follows: 0, less than 10% of positive cells; 1, 11–25%; 2, 26–50%; 3, 51–75%; 4, more than 75% of positive cells. The final staining score for each tissue was calculated by multiplying the staining intensity value by the percentage of positively stained cells, less than 3 points was considered negative(−), 3–6 points was considered weakly positive( +), 6–9 was considered positive (+ +); 9–12 points was considered strongly positive(+ + +)^42,43^.

### Annalysis of IL-6, IL-8 and IL-10 in serum

Peripheral blood samples (5 mL) were collected from all female dogs used in this study. The blood samples were centrifuged at 3500 rpm for 10 min to allow sedimentation of the blood cells. The serum was then divided into several portions and stored at – 80 °C for further experimentation. The serum IL-6, IL-8, and IL-10 levels were determined using the canine specific ELISAs kits (Jiancheng Biotechnology, Nanjing, China) according to the manufacturer’s instructions. The rang limits of detection for these assays are 2–600 ng/L, 5–1500 ng/L and 5–1500 ng/L for IL6, IL-8 and IL-10, respectively.

### Testing of IL-6, IL-8 and IL-10 protein in tissue using Western blot

The fifteen normal canine mammary gland and tumor tissue samples were lysed with RIPA lysis buffer (Beyotime Biotechnology, Shanghai, China), and the protein concentration was determined using a BCA protein assay kit (Beyotime Biotechnology, Shanghai, China). Equal amounts of protein lysate from all samples were separated on a 12–15% SDS-PAGE. Then, the separated proteins were transferred onto PVDF membranes. The blots were blocked with 5% skim milk at room temperature for 1 h and incubated overnight with primary antibodies against IL-6, IL-8 and IL-10 (Dilution, 1:500; Bioss Biotechnology, Beijing, China) at 4 °C. Then, the blots were incubated with HRP-conjugated goat anti-rabbit secondary antibody (ZSGB-BIO, Beijing, China; dilution, 1:2000) at 37 °C for 1 h. The blots were developed using ECL reagent (Tanon Bio, Shanghai, China), and the bands were photographed using a ChemidocXRS system (Bio-Rad, California, USA). β-actin (ZSGB-BIO, Beijing, China; dilution, 1:2000) was used as the loading control. Relative protein levels were quantified using ImageJ 1.48 software (National Institutes of Health, Bethesda, MD, USA).

### Statistical analysis

The data was analyzed using the SPSS 17.0 statistical software and GraphPad Prism version 5.0 (GraphPad Software Inc., San Diego, CA, USA). The results were expressed as mean ± standard deviation (SD). The differences in data groups showing normal distribution were assessed by One way-ANOVA followed by Turkey’s multiple comparison test or unpaired two-tailed *t* test. Kruskal Wallis test was used for non-normal distribution data. *P* < 0.05 was considered statistically significant. Chi-square test was used to analyze the correlation between the immunological indexes in each group (two variables).

## Supplementary Information

Supplementary Figure S1.

## Abbreviations

CMGTs: Canine mammary gland tumors
ER: Estrogen
PR: Progestin
Her-2: Human epidermal growth factor-2
IHC: Immunohistochemistry
ELISA: Enzyme-linked immunosorbent assay
SD: Standard deviation
