# Supplementary figures and images for: Expression and significance of IL-6 and IL-8 in canine mammary gland tumors
Source: Sci Rep. 2023 Jan 24;13:1302. doi: 10.1038/s41598-023-28389-3 (PMC9873921; doi:10.1038/s41598-023-28389-3)

Figure 3


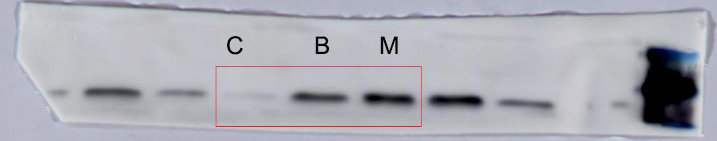


Note: C, Control; B, Benign; M, Malignant

IL-8

IL-10

IL-6

β-actin

Supplement: Supplementary file 1 — Supplementary Figure S1. [file 41598_2023_28389_MOESM1_ESM.docx]
